# Use of inhaled sevoflurane in adult survivors of cardiac arrest

**DOI:** 10.1186/2197-425X-3-S1-A746

**Published:** 2015-10-01

**Authors:** A Markota, B Kit, R Gagič, A Sinkovič

**Affiliations:** UKC Maribor, OIIM, Maribor, Slovenia

## Introduction

Use of inhalation anesthetic sevoflurane has some potential advantages over intravenous sedation in adult survivors of cardiac arrest. Wake-up times are shorter compared to intravenous sedation, neurologic assessment could potentially be performed sooner and there are reports of possible protection against ischaemic-reperfusion injury. There are no prospective studies or guidelines regarding use of sevoflurane in this setting.

## Objectives

Our aim is to present a case series of adult survivors of cardiac arrest who were sedated with sevoflurane.

## Methods

We performed a retrospective, single-centre study in a 12-bed tertiary medical intensive care unit.

## Results

Our cohort comprised 12 patients who were admitted from November 2014 - April 2015. Mean age was 63.9 ± 14.9 years, 9 (75%) were men. Mean time from collapse to return of spontaneous circulation was 13 ± 5 min. Mean duration of sedation with sevoflurane was 2.5 ± 2.4days. Five (42%) patients survived and 3 (25%) survived with Cerebral Performance Category 1 and 2. We recorded no self-extubations, one episode of unexpected awakening, no new onset hyperkalemia and no QT prolongation. Sevoflurane was discontinued in 2 (17%) patients because of ventilation problems. Sevoflurane was used as the only during therapeutic hypothermia in all patients and as the only during mechanical ventilation sedative in 6 (50%) patients. Propofol was used during mechanical ventilation simultaneously in 4 (33%) patients and midazolam in 2 (17%) patients. Transient increase in liver function tests was observed in 4 (33%) patients. In all cases it was attributed to ischaemic hepatitis and liver function normalized within a week in all patients. Renal failure developed in 2 (17%) patients and was attributed to circulatory shock in both patients.

## Conclusions

Sedation with sevoflurane in adult survivors of cardiac arrest was feasible and safe. Prospective trials are needed to evaluate the role of sedation with sevoflurane in this patient population.

